# The impact of Covid‐19 on innovation policies promoting Open Innovation

**DOI:** 10.1111/radm.12495

**Published:** 2021-07-23

**Authors:** Andrea S. Patrucco, Daniel Trabucchi, Federico Frattini, Jane Lynch

**Affiliations:** ^1^ Department of Marketing and Logistics College of Business Florida International University Miami FL USA; ^2^ School of Management Politecnico di Milano Milano Italy; ^3^ Department of Logistics and Operations Management Cardiff Business School Cardiff University Cardiff UK

## Abstract

Since the eruption of the Covid‐19 pandemic, in response to the global health emergency, governments have focused on designing policies aimed at the development of more innovative products and services. Effective collaboration, communication, and Open Innovation (OI) between government organizations, education and research institutions, and the marketplace have been fundamental to the success of each country's response during the crisis period. Using a comprehensive data set from OECD on innovation policies implemented by governments before and during the Covid‐19 crisis, this paper analyses the extent to which these innovation policies promote OI and how these policy decisions evolve to support an effective response to the pandemic. Through a cluster analysis, we identify four possible government innovation policy strategies (centralizers; conservative OI promoters; collaborative supporters; open collaborators) and analyze how these strategies evolve before and during Covid‐19. Our findings confirm that even though there is an increased use of innovation policies promoting OI during the crisis, there is little evidence of consistency between the policy strategy used pre‐Covid and during the crisis for each country. However, there is an increased use of four types of innovation policy instruments, i.e., those entailing formal consultation with stakeholders and experts; fellowships and postgraduate loans and scholarships; networking and collaborative platforms; and dedicated support to research infrastructures. Although the paper limits the scope of the analysis to the early government reactions in selected OECD countries, it captures an important moment in time (i.e., reaction to a severe shock), which opens avenues for future studies.

## 1 Innovation during the emergency period: an ‘open’ approach

The Covid‐19 health emergency has pushed society toward an unprecedented crisis. In such extraordinary circumstances, the urgency for mitigating the full impact of Covid‐19 by reducing its short and longer‐term impacts has driven governments to launch widescale and fast‐tracked innovation policies. This move is a complete shift in thinking with previous arguments that public organizations are not sufficiently innovative (Sørensen and Torfing, 2011). However, authors such as Azoulay and Jones (2020) emphasize that prevailing government attitudes to policymaking indicate that Covid‐19 can be beaten quickly by promoting innovation.

Initiatives such as hackathons, open research calls, financial support, funds to support the development of new technologies, and other process improvements are commonplace in UK and Europe. Specific governance structures are used to coordinate innovation through collaborative networks and joint innovation proposals (e.g., Ireland’s National Action Plan, Brazil’s MCTIC Virus network, and Canada Fonds de Recherche di Quebec Covid network). Joint calls for proposals supporting the later stages of the innovation process include the pioneering ‘Innovation for Italy’ program – this is a common platform where companies, universities, and research institutions are invited to contribute to the development and production of devices to prevent, diagnose, and monitor the spread of Covid‐19. In addition, the ‘Accelerating Covid‐19 Therapeutic Interventions and Vaccines’ (ACTIV) initiative involving the US National Institute of Health, the European Medicines Agency, and several biopharmaceutical companies speed up the research and development of effective Covid‐19 treatments and vaccines.

Since analyzing these initiatives in detail, in line with Chesbrough (2020), we note that common factors across governments and their innovation policy efforts include openness and collaboration.

Governments are reputed to embrace OI (e.g., Wang et al., 2012; Chesbrough and Vanhaverbecke, 2018; Jugend et al., 2020), though there is less knowledge about their use of collaborative policy instruments (UNECE, 2017). Studies show how OI can help respond to emergencies and crises (e.g., George et al., 2015; Zouraghi et al., 2018), though none of these crises studied have reached the scale of Covid‐19. This leads us to the following research questions:

RQ1: *To what extent did governments modify the use of existing innovation policies targeting external organizations in response to Covid‐19?*

RQ2: *What innovation policies promoting Open Innovation did governments use in response to Covid‐19?*

To address these questions, we adopt an exploratory approach. Using the Science, Innovation and Technology (STI) policy data published by the OECD (Organization for Economic Co‐operation and Development), we analyze the innovation policies with external organizations and the extent to what they promote OI of 44 countries in the 10 years before the Covid‐19 outbreak and during the emergency. Moreover, we explore which specific innovation policy instruments promoting OI have been more prevalent during the Covid‐19 crisis.

This analysis allows us to:

- compare the use of innovation policy strategies before and during an emergency period;
- identify whether there is a greater use of innovation policies promoting OI during the Covid‐19 crisis in comparison to normal times.

The results will contribute to the existing knowledge on innovation policies, which will be relevant for researchers and governments to guide the design of future innovation policies for better preparedness in a crisis.

## 2 Literature review

### 2.1 Open Innovation and its growing relevance in the public sector

The concept of OI was introduced by Chesbrough (2003), who first proposed the idea that firms can and should search for external sources of ideas and knowledge while fostering innovation. As traditional closed innovation approaches have become ineffective in addressing the emerging government policy challenges, a growing number of governments have tried to promote OI as part of their innovation strategies and policies (Bommert, 2010; Kankanhalli et al., 2017). OI has become an established and dominant paradigm in innovation management (Enkel et al., 2020) with two main trends identified: broadening definitions, and a move toward collaborative and integrative approaches.

OI definitions in the innovation management literature have broadened to become more inclusive, ‘*a distributed innovation process based on purposively managed knowledge flows across organizational boundaries*’ (Chesbrough and Bogers, 2014, p. 1). Best practices for implementing OI vary from more open and informal practices such as crowdsourcing and calls for ideas and proposals to more collaborative and integrated approaches such as joint‐ventures, consortia, and cross‐industries and university alliances (Felin and Zenger, 2014).

OI has become one of the most debated topics in innovation for researchers, professionals, and policymakers (Bogers et al., 2018). The diffusion of OI in the public context during the last decade has also raised the attention of academics from both innovation and public management fields, which is demonstrated by a growing number of publications in recent years (Jugend et al., 2020). OI practices in the public sector can be influenced and shaped by different goals, such as moving from human capital development, to fundraising and promoting cooperation and competition (Chesbrough and Vanhaverbecke, 2018). One of the distinguishing factors when applying OI in the public sector is its connection with government policies. This can be intended in two ways.

On the one hand, governments can work with each other to define OI policies which support entrepreneurship and help to improve their products and services to citizens (Mergel and Desouza, 2013). Although the diffusion of these initiatives is growing, public sector organizations are still early adopters of collaborative innovation models (Kankanhalli et al., 2017). They face several challenges connected to their implementation, particularly in the way that the ‘collaboration spirit’ can be introduced in an environment which is normally characterized by a lack of innovation culture (Pedersen, 2020). Consequently, it is not uncommon for public institutions to renounce the launch of innovation policies and initiatives due to these challenges.

On the other hand, to introduce product and service innovations that are of direct benefit to society and the local economy, governments can work to create and introduce policies that stimulate the use of OI with external organizations and individuals (De Jong et al., 2010). Governments need to engage in strategic collaborations with other organizations, research institutions, and even citizen networks to rapidly develop, test, and launch solutions for improving service performance and value creation (Lee et al., 2012; Gascò, 2017).

In this sense, Wang et al. (2012) grouped the public sector OI approaches into five areas – R&D; technology; infrastructure; region; and education. More recently, Jugend et al. (2020) identified four different dimensions of public policy support to innovation, i.e., financial support for R&D activities; development through innovation; support for sectorial programs; and university‐industry‐government collaboration.

Authors such as Leckel et al. (2020) have also acknowledged the absence of research exploring how OI can be better used as a strategic policy lever to promote business collaborations and growth. Leckel et al. argue there are clear reasons why applying OI at the local authority level brings important collaboration opportunities for smaller businesses leading to business growth – thus calling for stronger policy support.

As this research objective is to explore governments’ innovation policies during normal times and during the Covid crisis to stimulate the launch of new products and services which reduce the societal impact of the health emergency, our perspective on the application of OI in the public sector will fall in this second area to explore OI as a strategic policy lever for business collaboration and growth.

### 2.2 The role of Open Innovation for better emergency response

An emerging theory is that OI leads to high‐level impact and longer‐term outcomes regardless of whether private or public organizations shape the ‘innovation’ landscape. Building on existing knowledge that highlights the link between OI and social impact (Chesbrough and Di Minin, 2014), a special issue of the R&D Management Journal proposes that OI may be deliberately leveraged for improving societal outcomes (Ahn et al., 2019). In particular, many studies highlight the role of crowdsourcing to address societal problems (De Silva and Wright, 2019; Randhawa et al., 2019; Rayna and Striukova, 2019; Smart et al., 2019; Beck et al., 2020).

The literature also shows how OI might play a role in influencing societal issues, especially in hard times, like during crises, emergencies, or natural disasters. For example, OI has been studied as a moderator during the financial crisis (in 2008), evidencing how ‘openness’ may help a better recovery for companies following a financial shock (Yun et al., 2018). This is because OI allows firms to minimize the resource limitations and any risk surrounding innovation during the crisis (Zouraghi et al., 2018). Other studies show that firms adopt a wider use of OI strategies following the last financial crisis to seek exploitation of existing assets (Laperche et al., 2011).

Focusing on emergency management, the traditional literature places having dedicated structures to manage emergency situations as a central organizational need; nevertheless, the diffusion of digital technologies also encourages these organizational structures to be open and include contributions coming from many sources (Park and Johnston, 2018). Similarly, moving to the field of public‐private partnerships (PPP), Open Innovation and the ability to generate new business models and service platforms all emerged as key drivers to respond to medical emergencies (George et al., 2015).

In a similar perspective, there is a wide and growing literature on how citizens and local communities are willing to contribute to the recovery process generating spontaneous innovation (e.g., Shepherd and Williams, 2014). Contingent events, such as the recent Covid‐19 health emergency, elevate innovation to become a necessity for protecting society. These events demonstrate that, despite external pressures, public organizations can successfully overcome traditional innovation challenges and promote the implementation of OI practices successfully (Chesbrough, 2020). Even accepting and building citizens’ resilience becomes a form of action (Williams and Shepard, 2016).

To summarize this literature review, two points are particularly important. First, several OI scholars demonstrate that also governments and public entities can effectively rely on openness to foster and support innovation through the design of appropriate policies. Second, there is a growing body of literature that shows the role of OI during emergencies, crises, and disasters. However, there is a lack of studies integrating the two perspectives. The current pandemic represents a scenario where these two streams may be joined – we do this by addressing the research questions presented in the introduction, and studying if and how an emergency may impact the type and nature of innovation policies implemented by governments.

## 3 Research methodology

### 3.1 The EC/OECD framework for the classification of innovation policies

This study refers to ‘innovation policy’ in line with the definition provided by Edquist (2001, p. 18), who defines it as a ‘*public action that influences technical change and other kinds of innovations*’, which includes ‘*elements of R&D policy, technology policy, infrastructure policy, regional policy and education policy*’.

The EC/OECD STIP framework (EC/OECD, 2020a) is an internationally recognized standard that uses a functional approach to classify innovation policy instruments. The framework groups STI policies into five categories:

1. *Governance* – all government policies aimed to formalize a governance and a country‐innovation strategy that stimulates innovation research and development.
2. *Direct financial support* – government policies which provide direct economic impetus to support innovation research and development.
3. *Indirect financial support* – government policies aimed to provide indirect economic impetus for supporting innovation research and development.
4. *Collaborative infrastructures* – government policies aimed to ease collaboration and information sharing to stimulate innovation research and development.
5. *Guidance, regulation, and incentives* – government policies aimed to regulate innovation research and development activities.

The policies and the relative instruments for each group are presented in Table 1. We further adopt a two‐stage approach to the analysis of innovation policies, i.e., during normal times and during the Covid‐19 crisis.

**Table 1:** EC/OECD STI policy instrument taxonomy

Governance	Direct financial support	Indirect financial support	Collaborative infrastructures (soft and physical)	Guidance, regulation and incentives
National strategies, agendas, and plans Creation or reform of governance structure or public body Policy intelligence Formal consultation of stakeholders or experts Horizontal STI coordination bodies Regulatory oversight and ethical advice bodies Standards and certification for technology development and adoption Public awareness campaigns and other outreach activities	Institutional funding for public research Project grants for public research Grants for business R&D and innovation Centers of excellence grants Procurement programs for R&D and innovation Fellowships and postgraduate loans and scholarships Loans and credits for innovation in firms Equity financing Innovation vouchers	Corporate tax relief for R&D and innovation Tax relief for individuals supporting R&D and innovation Debt guarantees and risk‐sharing schemes	Networking and collaborative platforms Dedicated support to research infrastructures Information services and access to datasets	Technology extension and business advisory services Emerging technology regulation Labor mobility regulation and incentives Intellectual property regulation and incentives Science and innovation challenges, prizes and awards

### 3.2 Stage 1 – Innovation policies targeting external organizations and innovation policies promoting OI in normal times

We considered data from the STIP Compass Project (EC/OECD, 2020b), a joint initiative between the European Commission and the OECD. The data set includes qualitative (i.e., documents of innovation policies published by public organizations) and quantitative (i.e., through surveys collecting information on yearly budget ranges, count of policy instruments and responsible organizations, and key innovation policy metrics) data on STI policies for each of the OECD member countries starting from 1980, and it uses the EC/OECD STIP policy framework.

For the analysis, data are limited to 44 OECD countries (selecting those countries where data were available for analyses in both stages 1 and 2) between 2010 and 2020.

To analyze the innovation policy initiatives that target and involve external entities, we limit our search to two target groups: for‐profit (i.e., private companies and intermediaries) and research organizations (i.e., universities and other research institutions). We selected these groups because these are the most important actors acknowledged in the innovation literature as key players that public institutions involve when promoting OI (e.g., Lee et al., 2012).

Second, data were separated by policy groups (governance; direct financial support; indirect financial support; collaborative infrastructure; guidance, regulation, and incentives). This way, we are able to isolate all the innovation policies that, at country level, were specifically designed to stimulate innovation within these economic groups. These innovation policy groups include a broad set of instruments. It is worth noting at this stage that not all of them can be classified under the umbrella of OI.

For this reason, to identify the subset of these innovation policies that promote OI, we extracted the data by restricting the search to 11 policy instruments (in line with the definitions by Chesbrough and Crowther, 2006; Chesbrough and Schwartz, 2007; Pisano and Verganti, 2008; Chesbrough and Brunswicker, 2013) as follows, (i) Formal consultation with stakeholders and experts; (ii) Horizontal STI coordination bodies; (iii) Institutional funding for public research; (iv) Project grants for public research; (v) Grants for business and R&D and innovation; (vi) Centers for excellence and grants; (vii) Procurement programs for R&D and innovation; (viii) Fellowships and postgraduate loans and scholarships; (ix) Innovation vouchers; (x) Networking and collaborative platforms; (xi) Dedicated support to research infrastructures.

Using these data, each country is profiled in terms of:

- Total innovation policies during the period beginning 2010 – beginning of 2020 (i.e., pre‐Covid‐19).
- Total innovation policies targeting external organizations (i.e., firms, intermediaries, and research institutions) during the period beginning 2010 – beginning 2020, segmented into the policy categories of the OECD‐STIP framework (i.e., governance, direct financial support, indirect financial support, collaborative infrastructure, guidance, regulation, and incentives).
- Total innovation policies targeting external organizations promoting OI during the period beginning 2010 – beginning 2020.
- R&D performance, such as the Gross Domestic R&D expenditure (GERD), the high technology exports, the triadic patent families, and the number of researchers.

We perform a two‐step clustering analysis (e.g., Okazaki, 2006) to characterize the STI innovation policies model adopted by the countries included in the dataset. This first analysis has the objective of positioning each government in terms of its strategic orientation to use policy instruments targeting external organizations, and policies promoting OI. Stage 1 of the analysis is an important prerequisite for Stage 2.

### 3.3 Stage 2 – Innovation policies targeting external organizations and innovation policies promoting OI during Covid‐19

We compare government behavior during normal times and during the Covid‐19 emergency period, including innovation policies that target external organizations and innovation policies targeting external organizations promoting OI.

To achieve this, we continue to use the data collected through the STIP Compass project but included in the STIP Covid‐19 tracker (OECD, 2021). This tracker uses a similar structure of data on innovation policies during normal times, but with a specific focus on policy responses to Covid‐19. The OECD elaborated these data through the information collected from the survey ‘STI Policy response to Covid‐19’ (Appendix A) and a systematic analysis of government public documents between February and December 2020 (so, during Covid‐19).

To evaluate the effectiveness of these policies, we further complement this information using data released by the OECD Observatory of Public Sector Innovation (OPSI, 2020). Through their Covid‐19 innovative response tracker, the OECD benefits from up‐to‐date information about the number and types of innovations introduced by each country during the Covid‐19 crisis (see Appendix B for more details).

Adopting a similar approach to Stage 1, we perform a two‐step clustering analysis to characterize the innovation policies implemented in response during Covid‐19. This second analysis allows us to evaluate the policy response from each country included in the dataset and to identify if any differences are present compared with the policy approach in normal times, especially the inclusion of policies promoting OI, among others. IBM SPSS 26.0 was used to perform the analyses.

## 4 Results

### 4.1 Innovation policies: an overview in business‐as‐normal times

Table 2 reports each country's main descriptive results for innovation policies targeting external organizations in normal times.

**Table 2:** Innovation policy response in main OECD countries (2010–2020 period) and main R&D indicators (authors’ elaboration from OECD data)

	Total innovation policies initiatives	Total innovation policies with external organizations		Innovation policies using Governance instruments	Innovation policies using Direct financial support instruments	Innovation policies using indirect financial support instruments	Innovation policies using collaborative infrastructure STI instruments	Innovation policies using guidance, regulation, incentives instruments	Total innovation policies with external organizations promoting OI^a^		Gross Domestic R&D expenditure	High technology exports	Triadic patent families	Researchers
Argentina	34	20	58.82%	45.00%	50.00%	0.00%	25.00%	10.00%	16	80.0%	1.88	12.79	0.28	9.03
Australia	115	74	64.35%	62.16%	45.95%	2.70%	21.62%	8.11%	47	63.5%	1.88	12.79	0.28	9.03
Austria	125	74	59.20%	39.19%	44.59%	1.35%	18.92%	10.81%	41	55.4%	3.16	9.78	0.99	10.36
Belgium	146	78	53.42%	34.62%	47.44%	3.85%	17.95%	6.41%	43	55.1%	2.6	9.49	0.85	11.96
Brazil	58	46	79.31%	41.30%	34.78%	2.17%	30.43%	2.17%	26	56.5%	1.15	12.28	0.01	1.49
Canada	89	69	77.53%	34.78%	30.43%	0.00%	26.09%	15.94%	34	49.3%	1.55	12.85	0.35	8.41
Chile	38	32	84.21%	31.25%	50.00%	0.00%	18.75%	3.13%	23	71.9%	0.36	6.08	0.03	1.11
China	24	17	70.83%	29.41%	23.53%	5.88%	47.06%	23.53%	10	58.8%	2.13	23.81	0.16	2.24
Colombia	90	73	81.11%	47.95%	41.10%	4.11%	30.14%	20.55%	44	60.3%	0.24	8.69	0.01	0.25
Costa Rica	43	19	44.19%	47.37%	36.84%	0.00%	10.53%	15.79%	8	42.1%	0.57	18.26	0.01	1.33
Czech Republic	39	33	84.62%	39.39%	39.39%	6.06%	12.12%	18.18%	20	60.6%	1.79	12.77	0.1	7.35
Denmark	55	37	67.27%	45.95%	21.62%	2.70%	24.32%	10.81%	17	45.9%	3.06	11.56	1.02	15.48
Estonia	50	32	64.00%	50.00%	37.50%	3.13%	12.50%	12.50%	17	53.1%	1.29	16.06	0.27	7.4
Finland	75	49	65.33%	51.02%	34.69%	0.00%	20.41%	4.08%	27	55.1%	2.76	7.75	1.23	14.55
France	96	62	64.58%	43.55%	35.48%	1.61%	9.68%	12.90%	24	38.7%	3.02	13.67	1.22	9.34
Germany	116	95	81.90%	40.00%	67.37%	1.05%	17.89%	4.21%	94	98.9%	3.02	13.67	1.22	9.34
Greece	46	32	69.57%	31.25%	34.38%	9.38%	12.50%	18.75%	13	40.6%	1.13	10.36	0.09	8.44
Iceland	20	14	70.00%	50.00%	7.14%	7.14%	21.43%	7.14%	5	35.7%	2.13	13.78	0.16	11.57
India	8	8	100.00%	75.00%	0.00%	0.00%	0.00%	25.00%	5	62.5%	0.62	7.01	0.04	0.58
Ireland	79	36	45.57%	27.78%	52.78%	0.00%	8.33%	11.11%	20	55.6%	1.05	21.45	0.34	9.28
Israel	57	42	73.68%	28.57%	47.62%	2.38%	21.43%	14.29%	27	64.3%	4.54	13.04	1.55	17.43
Italy	123	65	52.85%	50.77%	26.15%	13.85%	12.31%	6.15%	26	40.0%	1.35	6.84	0.36	5.43
Japan	54	33	61.11%	27.27%	60.61%	0.00%	9.09%	0.00%	27	81.8%	3.2	13.81	3.34	10.01
Korea	78	61	78.21%	59.02%	19.67%	0.00%	16.39%	21.31%	22	36.1%	4.55	14.18	1.48	14.43
Latvia	64	45	70.31%	35.56%	46.67%	4.44%	11.11%	6.67%	25	55.6%	0.51	16.63	0.03	3.93
Luxembourg	**42**	27	64.29%	22.22%	48.15%	0.00%	29.63%	14.81%	17	63.0%	1.26	6.71	0.32	6.31
Lithuania	116	92	79.31%	32.61%	44.57%	1.09%	13.04%	10.87%	37	40.2%	0.88	11.71	0.08	6.42
Mexico	12	6	50.00%	0.00%	83.33%	16.67%	16.67%	33.33%	5	83.3%	0.49	15.17	0.01	0.82
New Zealand	77	48	62.34%	18.75%	33.33%	2.08%	27.08%	25.00%	27	56.3%	1.23	8.58	0.6	7.94
Netherlands	67	43	64.18%	44.19%	39.53%	2.33%	25.58%	2.33%	27	62.8%	1.99	18.58	1.49	9.38
Norway	67	37	55.22%	51.35%	32.43%	0.00%	13.51%	5.41%	17	45.9%	2.11	18.43	0.3	12.3
Peru	36	12	33.33%	66.67%	25.00%	8.33%	8.33%	8.33%	4	33.3%	0.12	4.66	0.01	0
Poland	130	82	63.08%	31.71%	59.76%	2.44%	6.10%	6.10%	52	63.4%	1.03	7.74	0.08	5.93
Portugal	155	112	72.26%	45.54%	34.82%	4.46%	19.64%	6.25%	60	53.6%	1.32	4.98	0.08	9.23
Russian Federation	97	82	84.54%	39.02%	30.49%	4.88%	17.07%	14.63%	44	53.7%	1.11	11.52	0.02	5.69
Slovenia	77	61	79.22%	37.70%	44.26%	0.00%	16.39%	4.92%	35	57.4%	1.85	6.24	0.19	9.24
South Africa	52	36	69.23%	52.78%	27.78%	0.00%	25.00%	0.00%	20	55.6%	0.8	4.64	0.03	1.66
Spain	98	50	51.02%	16.00%	68.00%	2.00%	6.00%	10.00%	36	72.0%	1.2	7.05	0.14	6.84
Sweden	45	29	64.44%	34.48%	48.28%	3.45%	34.48%	3.45%	20	69.0%	3.33	13.22	1.29	15.04
Switzerland	53	24	45.28%	41.67%	45.83%	8.33%	4.17%	8.33%	13	54.2%	3.37	11.4	2.27	8.94
Thailand	83	70	84.34%	35.71%	45.71%	11.43%	25.71%	32.86%	37	52.9%	0.62	21.51	0.01	1.5
Turkey	117	85	72.65%	41.18%	43.53%	3.53%	5.88%	9.41%	39	45.9%	0.96	2.53	0.02	4.01
United Kingdom	114	78	68.42%	41.03%	30.77%	3.85%	17.95%	12.82%	33	42.3%	1.66	21.07	0.68	9.04
United States	95	58	61.05%	50.00%	29.31%	0.00%	51.72%	34.48%	41	70.7%	2.79	13.82	0.81	8.93

Data as of December 2020. The table includes information about the OECD countries for which data is available for both pre and during Covid‐19 (44 of the 56 OECD countries).

^a^ as a % of total innovation policies with external organizations. The total can be higher than 100% as innovation policies can be classified in more than one category.

^b^ as a percentage of GDP.

^c^ as% of manufactured exports.

^d^ per GDP billion USD PPPs.

^e^ per thousand employment.

Cluster analysis is performed using as input variables the ‘total innovation policies with external organizations’ and the ‘total innovation policies with external organizations promoting OI’ (noted as percentages in columns 2 and 8 in Table 2).

As illustrated in Table 3, the procedure identifies four robust clusters (Silhouette coefficient of 0.72; Rousseeuw, 1987), which can be differentiated according to the two clustering variables used.

**Table 3:** Cluster analysis for innovation policies in main OECD countries (2010–2020 period; average values per cluster reported)

	*Centralizers (N = 12)*	*Conservative OI promoters (N = 10)*	*Collaborative supporters (N = 14)*	*Collaborators (N = 8)*	*P*‐value
Clustering input variables					
Total innovation policies with external organizations	53.78%	60.23%	74.50%	81.95%	0.000***
Total innovation policies with external organizations promoting OI	48.74%	70.95%	47.41%	66.84%	0.000***
Types of policy instruments					
Governance	43.6%	33.3%	41.8%	41.2%	0.071^NS^
Direct financial support	37.7%	53.3%	32.3%	39.2%	0.042*
Indirect financial support	3.5%	3.0%	3.9%	2.4%	0.31^NS^
Collaborative infrastructure	13.6%	22.6%	19.0%	20.5%	0.048*
Guidance, regulation, and incentives	10.6%	12.3%	12.1%	14.2%	0.247^NS^
R&D outcomes					
GERD (as a % of GDP)	1.8188	1.905	1.5307	1.8858	0.493^NS^
High technology exports (as a % of manufacturing exports)	12.1975	12.168	12.1143	11.4137	0.825^NS^
Triadic patent families (per GDP billion USD PPPs)	0.7042	0.804	0.29	0.4125	0.71^NS^
Researchers (per thousand employment)	8.2358	8.132	7.2357	5.9425	0.263^NS^

*** *P* < 0.001,

** *P* < 0.01,

* *P* < 0.05,

^NS^ *P* > 0.05.

After the ANOVA procedure verification, we note these clusters have marginal differences in terms of the type of innovation policies implemented.

**Figure 1:**
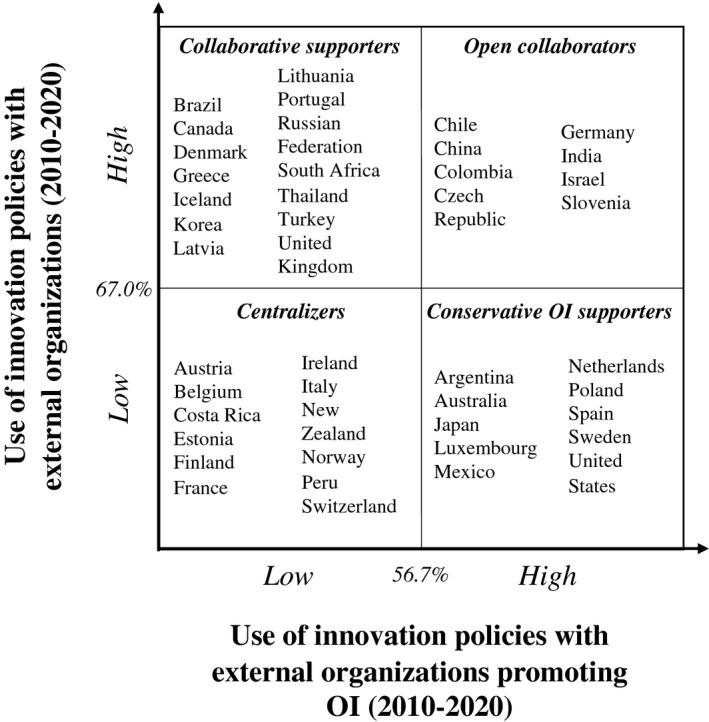
Government strategies to innovation policies with external organizations during 2010–2020 (*Note*: 67.0% and 56.7% represent the average value for the clustering variables for the countries included in the analysis).

Figure 1 shows how countries included in the dataset are mapped into the four clusters. Each quadrant represents a different innovation policy strategy based on the relative use of innovation policies with external organizations and those promoting OI.

12 OECD countries adopt what we call the *centralizers* strategy, characterized by less intense use of innovation policies targeting external organizations and those that promote OI. These are governments that mostly focus their innovation policy systems targeting internal governmental entities and economic actors, using for the large part ‘closed’ regulatory instruments, related to the identification of national innovation strategies and agendas, the definition of policy intelligence, and formalization of regulation related to technology standards and certifications, and intellectual property.

10 OECD countries adopt what we call the *conservative OI promoters* strategy, characterized by a low use of innovation policies with external organizations, but several of these promote OI. These governments adopt a focused approach in the use of OI promoting instruments. They mainly use direct financial support tools; for these reasons, the most adopted policy instruments fall within the category of economic support to R&D research grants in different forms.

14 OECD countries adopt what we call the *collaborative supporters*’ strategy, characterized by a significantly high use of innovation policies with external organizations, but few of these promote OI. These governments implement innovation policies mostly regulatory in nature designed to target external entities, such as firms, higher education institutions, research institutes, industry associations, incubators, and technology transfer offices.

8 OECD countries adopt what we call the *open collaborators* strategy, characterized by a significantly high use of innovation policies with external organizations, and most of these also promote OI. These governments focus their innovation policy systems targeting external organizations, through instruments such as horizontal innovation coordination bodies, the establishment of networking and collaborative platforms, and budget to sponsor R&D research grants in different forms.

### 4.2 Innovation policies with external organizations during Covid‐19

The first stage of the cluster analysis reveals different approaches adopted by governments seeking to promote innovation. Only 50% of the countries make high use of innovation policies with external organizations (31.8% as *collaborative supporters* and 18.2% as *open collaborators*). This percentage decreases to 40% if we look at those countries adopting innovation policies with external organizations promoting OI (22.7% as *conservative OI supporters* and 18.2% as *open collaborators*).

Table 4 reports each country's main descriptive results during the Covid‐19 period to understand whether the situation changes.

**Table 4:** Innovation policy response during Covid‐19 (authors’ elaboration from OECD and OPSI data)

	Total STI policy initiatives	Total innovation policies with external organizations		Innovation policies using Governance instruments	Innovation policies using Direct financial support instruments	Innovation policies using indirect financial support instruments	Innovation policies using collaborative infrastructure STI instruments	Innovation policies using guidance, regulation, incentives instruments	Total innovation policies with external organizations promoting OI^a^		Number of innovations
Argentina	12	6	50.0%	16.7%	83.3%	0%	33.3%	16.7%	5	83.3%	2
Australia	17	6	35.3%	33.3%	83.3%	0%	16.7%	0.0%	6	100.0%	9
Austria	12	9	75.0%	0.0%	88.9%	0%	11.1%	0.0%	8	88.9%	30
Belgium	26	12	46.2%	16.7%	58.3%	0%	33.3%	0.0%	9	75.0%	2
Brazil	13	6	46.2%	0.0%	83.3%	0%	50.0%	0.0%	5	83.3%	11
Canada	57	22	38.6%	13.6%	59.1%	0%	13.6%	13.6%	16	72.7%	10
Chile	13	10	76.9%	30.0%	30.0%	0%	40.0%	10.0%	6	60.0%	0
China	16	10	62.5%	30.0%	10.0%	20%	10.0%	20.0%	2	20.0%	6
Colombia	5	2	40.0%	100.0%	50.0%	0%	0.0%	0.0%	1	50.0%	3
Costa Rica	10	7	70.0%	0.0%	85.7%	0%	14.3%	28.6%	6	85.7%	1
Czech Republic	15	9	60.0%	22.2%	77.8%	0%	0.0%	0.0%	7	77.8%	16
Denmark	9	2	22.2%	50.0%	50.0%	0%	0.0%	0.0%	1	50.0%	5
Estonia	9	1	11.1%	0.0%	100.0%	0%	0.0%	0.0%	1	100.0%	10
Finland	29	11	37.9%	18.2%	54.5%	9%	9.1%	0.0%	6	54.5%	5
France	15	7	46.7%	14.3%	57.1%	0%	42.9%	0.0%	6	85.7%	12
Germany	21	13	61.9%	7.7%	69.2%	15%	23.1%	15.4%	10	76.9%	5
Greece	18	12	66.7%	0.0%	83.3%	0%	16.7%	0.0%	12	100.0%	5
Iceland	3	3	100.0%	33.3%	66.7%	0%	0.0%	0.0%	3	100.0%	1
India	2	0	0.0%	0.0%	0.0%	0%	0.0%	0.0%	0	0.0%	7
Ireland	8	5	62.5%	20.0%	40.0%	0%	20.0%	20.0%	3	60.0%	14
Israel	5	2	40.0%	0.0%	100.0%	0%	0.0%	0.0%	2	100.0%	5
Italy	8	5	62.5%	40.0%	40.0%	0%	20.0%	0.0%	2	40.0%	5
Japan	13	13	100.0%	15.4%	53.8%	0%	38.5%	7.7%	8	61.5%	2
Korea	26	16	61.5%	25.0%	18.8%	0%	50.0%	18.8%	7	43.8%	9
Latvia	8	4	50.0%	25.0%	25.0%	0%	0.0%	50.0%	2	50.0%	6
Lithuania	14	8	57.1%	50.0%	62.5%	0%	25.0%	0.0%	0	*0.0%*	0
Luxembourg	1	0	0.0%	0.0%	0.0%	0%	0.0%	0.0%	0	0.0%	18
Mexico	6	4	66.7%	25.0%	75.0%	0%	0.0%	0.0%	3	75.0%	12
Netherlands	10	3	30.0%	0.0%	0.0%	100%	0.0%	0.0%	0	0.0%	3
New Zealand	11	7	63.6%	42.9%	85.7%	0%	0.0%	0.0%	6	85.7%	5
Norway	25	21	84.0%	9.5%	66.7%	0%	23.8%	4.8%	14	66.7%	3
Peru	6	1	16.7%	0.0%	100.0%	0%	0.0%	0.0%	1	100.0%	1
Poland	17	8	47.1%	0.0%	87.5%	0%	12.5%	0.0%	7	87.5%	1
Portugal	17	9	52.9%	11.1%	66.7%	0%	33.3%	0.0%	7	77.8%	37
Russian Federation	27	21	77.8%	28.6%	52.4%	29%	0.0%	9.5%	10	47.6%	1
Slovenia	14	11	78.6%	18.2%	45.5%	0%	18.2%	0.0%	6	54.5%	4
South Africa	15	2	13.3%	50.0%	50.0%	0%	0.0%	0.0%	2	100.0%	1
Spain	21	13	61.9%	30.8%	53.8%	0%	15.4%	7.7%	9	69.2%	8
Sweden	14	12	85.7%	8.3%	83.3%	0%	8.3%	0.0%	10	83.3%	2
Switzerland	20	8	40.0%	12.5%	37.5%	0%	50.0%	0.0%	3	37.5%	3
Thailand	6	2	33.3%	0.0%	100.0%	0%	0.0%	0.0%	2	100.0%	0
Turkey	8	3	37.5%	0.0%	66.7%	0%	66.7%	0.0%	2	66.7%	1
United Kingdom	18	3	16.7%	33.3%	100.0%	0%	0.0%	0.0%	3	100.0%	23
United States	40	29	72.5%	24.1%	55.2%	0%	24.1%	13.8%	19	65.5%	22

Data as of December 2020. The table includes information about the OECD countries for which data is available for both pre and during Covid‐19 (44 of the 56 OECD countries).

^a^ as a % of total innovation policies with external organizations during Covid‐19. The total can be higher than 100% as innovation policies can be classified in more than one category.

To corroborate the previous findings, we performed a second cluster analysis using input variables, the ‘total innovation policies with external organizations’, and the ‘total innovation policies with external organizations promoting OI’ during the Covid‐19 period (shown as percentages in columns 2 and 8 in Table 4).

The procedure identifies again four robust clusters (Silhouette coefficient of 0.67), which can be differentiated according to the two clustering variables used (Table 5).

**Table 5:** Cluster analysis for innovation policies in main OECD countries (Covid‐19 period; average values per cluster reported)

	*Centralizers (N = 9)*	*Conservative OI promoters (N = 13)*	*Collaborative supporters (N = 11)*	*Collaborators (N = 11)*	*P*‐value
Clustering input variables					
Total innovation policies with external organizations	28.63%	33.93%	72.36%	69.49%	0.000^***^
Total innovation policies with external organizations promoting OI	34.30%	91.35%	47.24%	83.67%	0.000^***^
Types of policy instruments					
Governance	22.9%	13.7%	26.4%	16.5%	0.044^*^
Direct financial support	31.5%	81.7%	43.2%	76.0%	0.012^*^
Indirect financial support	12.1%	0.0%	4.4%	1.4%	0.038^*^
Collaborative infrastructure	14.0%	15.6%	24.5%	11.1%	0.102^NS^
Guidance, regulation, and incentives	5.6%	2.3%	9.5%	4.7%	0.288^NS^
Outcomes					
Number of innovations	5.67	7.1	6	11.1	0.041^*^

*** *P* < 0.001,

** *P* < 0.01,

* *P* < 0.05,

^NS^ *P* > 0.05.

Following the ANOVA procedure verification, we note that these clusters show greater differences in terms of the type of policies used than during the normal pre‐Covid period. The groups of *centralizers* and *collaborative supporters* make a significantly higher use of governance policy instruments than other clusters. However, the *conservative OI promoters* and the *collaborators* groups significantly higher use of direct financial support instruments than other clusters. Countries classified as *centralizers* also make a significantly higher use of indirect financial instruments compared to other groups.

The most interesting result is found in Table 5 concerning the OI outcome. Countries in the *collaborators* cluster have introduced a significantly higher number of innovations than other groups. The second‐highest value is for the *conservative OI promoters*, which still includes countries making high use of policies promoting OI.

**Figure 2:**
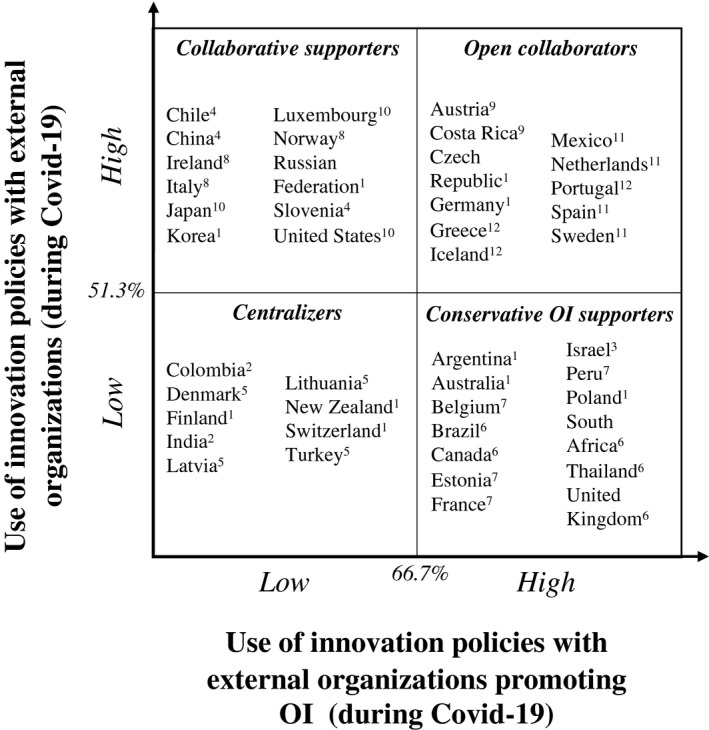
Government strategies to innovation policies with external organizations during Covid‐19 (*Note*: 51.3% and 66.7% represent the average value for the clustering variables for the countries included in the analysis). ^1^No variation of innovation policy strategy compared to normal times; ^2^From Open collaborators to Centralizers; ^3^From Open collaborators to Conservative OI supporters; ^4^From Open collaborators to Collaborative supporters; ^5^From Collaborative supporters to Centralizers; ^6^From Collaborative supporters to Conservative OI supporters; ^7^From Centralizers to Conservative OI supporters; ^8^From Centralizers to Collaborative supporters; ^9^From Centralizers to Open collaborators; ^10^From Conservative OI supporters to Collaborative supporters; ^11^From Conservative OI supporters to Open collaborators; ^12^From Collaborative supporters to Open collaborators.

Following this second analysis, countries are positioned in each innovation policy strategy cluster, using the same matrix as stage 1. The second matrix is represented in Figure 2, together with the details about quadrant changes compared to the previous matrix. Several governments change their innovation policy strategy during Covid‐19 compared to the normal period. The implications of these results are discussed in the next section.

To further understand the natures of these changes from an OI perspective, we finally compare the variations in terms of innovation policy instruments with external organizations promoting OI before and during Covid‐19 (Table 6).

**Table 6:** Innovation policy instruments with external organizations promoting OI before and during Covid‐19

	*2010‐2020*		*During COVID‐19*		*P*‐value
Total innovation policies with external organizations	2,178		358		
Total innovation policies with external organizations promoting OI	1,530	*70.25%*	288	*80.45%*	0.014^*^
Formal consultation with stakeholders and experts	98	*4.50%*	6	*1.68%*	0.023^*^
Horizontal STI coordination bodies	49	*2.25%*	9	*2.51%*	0.207^NS^
Institutional funding for public research	141	*6.47%*	55	*15.36%*	0.011^*^
Project grants for public research	317	*14.55%*	91	*25.42%*	0.0071^**^
Grants for business and R&D and innovation	318	*14.60%*	62	*17.32%*	0.094^NS^
Centers for excellence and grants	61	*2.80%*	5	*1.40%*	0.178^NS^
Procurement programs for R&D and innovation	45	*2.07%*	17	*4.75%*	0.056^NS^
Fellowships and postgraduates loans and scholarships	64	*2.94%*	2	*0.56%*	0.006^**^
Innovation vouchers	41	*1.88%*	1	*0.28%*	0.053^NS^
Networking and collaborative platforms	262	*12.03%*	30	*8.38%*	0.047^*^
Dedicated support to research infrastructures	134	*6.15%*	10	*2.79%*	0.028^*^

*** *P* < 0.001,

** *P* < 0.01,

* *P* < 0.05,

^NS^ *P* > 0.05.

While for some instruments there was no significant variation in use before and during Covid‐19, our analysis indicates a significant decrease in the use of four instruments during the Covid‐19 crisis period (i.e., formal consultation with stakeholders and experts; fellowships and postgraduates loans and scholarships; networking and collaborative platforms; and dedicated support to research infrastructures), and a notable increase in the use of two (i.e., institutional funding for public research; and project grants for public research). Most importantly, results show a significant increase (from 70.25% to 80.45%) of innovation policies promoting OI.

## 5 Discussion

The analysis confirms that during the Covid‐19 emergency, there is a more intense use of innovation policies promoting OI by governments. This finding aligns with the point made by previous scholars (e.g., George et al., 2015; Gascò, 2017). During these emergency periods, and even more urgently with Covid‐19, innovative solutions need to be developed and deployed quickly, and this forces governments to push different actors to collaborate more intensively.

### 5.1 How did innovation policy strategies change during Covid‐19?

Numerically, by comparing the distribution in the matrices in Figures 1 and 2, the number of countries making higher use of innovation policies promoting OI (*conservative OI promoters* and *collaborators*) increases from 18 (40%) to 24 (54.5%). In all 24 countries, a common characteristic is the presence of instruments to establish formal governance of innovation during Covid‐19, with the responsibility assigned to specific departments (e.g., Education, Science, and Research Ministry in Austria), or to ‘ad‐hoc’ task forces (like the ‘Research and innovation for preventing Coronavirus in Europe’ group in Portugal).

Only 10 countries did not experience any cluster change; of these, 4 were already characterized by their intense use of innovation policies promoting OI (Czech Republic, Germany, Korea, Russian Federation). In Germany, there have been multiple hackathons to stimulate innovative responses,^1^ paired with several open calls and challenges from the German Research Foundation for interdisciplinary research into epidemics and pandemics.^2^ In Russia, several measures were implemented to support SME research and development, both in terms of direct and indirect financial support.^3^

During the Covid‐19 crisis, 4 countries (Columbia, India, Austria, and Costa Rica) completely revolutionize their approach to innovation policies with external organizations. On the one hand, Colombia and India moved from being *collaborators* to *centralizers*, thus decreasing the use of both aspects. On the other, Austria and Costa Rica experienced the opposite path, adopting the *collaborators* strategy. These countries are well‐known in the news for several important innovation efforts. In Austria, an Epidemiological Reporting System^4^ which consolidates testing results and thus provides real‐time information about the extent of the pandemic in the country was launched (and replicated in other European countries), while the City of Vienna has created a ‘Homecare’ app to support patients and potentially infected to be digitally monitored in their homes.^5^ Costa Rica was often referred to by the media as a country that is providing an unprecedented innovation effort during the Covid‐19 period, especially noting the effective collaboration between public and private sectors.^6^

6 other countries (Chile, China, Slovenia, Japan, Luxembourg, and United States) experienced a decrease in the use of innovation policies with external organizations promoting OI (thus ending up in the *collaborative supporters* strategy quadrant).

Chile, China, and Slovenia decrease the promotion of OI in their policies but kept a high use of innovation policies with external organizations. Instead, Japan, Luxembourg, and United States decreased the use of policies promoting OI but increased the relative amount of innovation policies with external organizations. These countries focused their response more on the definition of an innovation governance in response to the emergency and more regulatory aspects. In China, efforts were made to introduce procedural and process innovations, to make procurement processes more flexible, and to support innovation in SMEs.^7^ This does not suggest that remarkable OI policies were not present in these countries. In the United States, the US National Institutes of Health launched a US$500m challenge to develop rapid coronavirus testing technologies, as well as an open call for a public‐private partnership to develop an international strategy for a coordinated research response to the pandemic with leading biopharmaceutical companies.^8^

The remaining 12 countries (other than Austria and Costa Rica) were characterized by an increase of policies promoting OI (ending up in the *conservative OI supporters* strategy quadrant).

In Brazil, Canada, South Africa, Thailand, and United Kingdom, this was made with a relative reduction of the use of innovation policies with external organizations compared to normal times. In Belgium, Estonia, France, and Peru, initial low use of innovation policies with external organizations changed using a more ‘focused effort’. They recognized the value of the engagement with external organizations and focused policies on the most complex (but also with the highest potential return) instruments. Through IT collaborations, the Australian and the UK Governments launched a new application and WhatsApp chat feature to help keep citizens informed about the crisis.^9^ The Brazilian government developed a specific app for communicating important messages to its citizens.^10^ The UK Chancellor offered a billion‐pound package of support exclusively to firms wishing to research and develop innovative solutions for Covid‐19.^11^ From this fund, further financial support was being offered to rescue technology firms and to ‘Future Fund’ business startups.

For Greece, Iceland, and Portugal, the increase in the use of policies promoting OI happened by maintaining a high use of innovation policies with external organizations (thus moving toward the *open collaborators* strategy quadrant). The Greek Ministry of Digital Governance issued a ‘Rapid Implementation of Mature Digi‐Tech Strategies’ call^12^ to accelerate the implementation of available technological solutions ready for quick implementation. The National Innovation Agency of Portugal provided reimbursable support and funding^13^ for the immediate development of relevant innovation projects that can help meet medical needs as well as an R&D incentive for relevant pre‐commercial projects. Portugal also issued a call ‘Doctorates 4 COVID‐19’ to fund 50 PhD scholarships on research relating to the pandemic^14^ and defined a mobilization plan to make the shift to working from home for public professionals easier.^15^

Finally, 11 countries did not modify their use of innovation policies promoting OI. Of these, 5 countries (Israel, Mexico, Netherlands, Spain, and Sweden) have maintained a high use – and decreased or increased the relative use of innovation policies with external organizations (moving from being *conservative OI promoters* to *collaborators*, or vice versa).

### 5.2 The nature and impact of innovation policies with external organizations promoting OI during Covid‐19

The analysis confirms that during Covid‐19, governments launched innovation policies characterized by an increased use of those promoting OI in response to the emergency, and no particular pattern was found compared with the strategy adopted during normal (pre‐Covid) times. In particular, the data confirms the prominent role of an OI approach during emergencies. For most countries, a great percentage of the innovation policies are made of policies that promote OI.

Direct financial support and collaboration infrastructure instruments are the most applied innovation policies for emergency management targeting external organizations. These include creating emergency funds for innovation development, establishing research grants, and creating horizontal collaborations with other countries and organizations. Special attention seems to be given to collaborative infrastructure policies, though data show a decrease in numbers compared to business‐as‐normal. Four instruments seem to have the most use during Covid‐19.

Among the innovation policy instruments promoting OI, our analysis highlights that four, in particular, experienced an increase in use.

The increase in the use of *formal consultation of stakeholders or experts* is directly connected with the need for a clear governance, which is well suited to the technical nature that any emergency brings, and stakeholders or experts require specific knowledge to join the public discussion.

For *fellowships and postgraduate loans and scholarships*, the result partially contrasts with the previous results about OI adoption in the public sector (e.g., Jugend et al., 2020). These instruments of direct financial support are in fact presented as more long‐term and riskier innovation policies, so not fully suitable for an immediate response. However, governments increased the use of these instruments so they can be considered valuable from a short‐term, fast‐response perspective.

Finally, *networking and collaborative platforms* and *dedicated support to research infrastructures* are correlated instruments, as they are both intended to design the necessary collaborative infrastructure for effective networking.

*Collaborative platforms* (such as the use of crowdfunding; Mejia et al., 2019) are already proven to be highly effective in emergency situations and play an important role in every‐day life (e.g., Cusumano et al., 2019); but according to our results, they can also contribute to favor innovation development during emergencies, as they are able to better connect individuals.

As a final note, our results show that countries that kept (or switched toward) a strategy with high use of policies promoting OI during Covid‐19 benefited from a higher number of innovations. This suggests that countries promoting OI were more effective in their responses to the Covid‐19 crisis. Still, there is considerable variety in the types of innovation policies with external organizations that are being employed to respond to the Covid‐19 emergency. This confirms that, during these emergency periods, innovative solutions need to be accessed quickly, forcing governments to deregulate and open their traditional boundaries, so that they may collaborate more intensively with external actors.

Although evaluating the effectiveness and value of these innovations is not possible in an objective way, this exploratory finding supports the idea that an ‘open’ approach is able to guarantee a better outcome in the public sector – at least in terms of volume.

## 6 Conclusions

### 6.1 Academic contributions

This research paper examines the characteristics of the government innovation policy strategies for 44 OECD countries during normal times and during Covid‐19 by analyzing the extent to which innovation policies were implemented to promote OI during a global emergency.

From an academic perspective, this research brings together two literature streams: focusing on the use of OI in the public sector (e.g., Chesbrough and Vanhaverbecke, 2018; Jugend et al., 2020; Leckel et al., 2020) and focusing on the role of OI in emergencies, crises and natural disasters (e.g., Shepherd and Williams, 2014; Park and Johnston, 2018; Zouraghi et al., 2018). The Covid‐19 pandemic and the OECD data represent a global case that enables us to assess the public reactions by using innovation policies promoting OI during an emergency that affected the entire globe.

Although exploratory in nature, this research offers an empirical examination of the diffusion and adoption of innovation policies promoting OI during crises events (with Covid‐19 used as the representation of a unique global emergency). We find that the government’s tendency to promote OI increases during these periods and, interestingly, a clear connection with the innovation policy strategy used during normal times does not seem to be present (i.e., changes of quadrants from Figures 1, 2 happen with no univocal trends). Nevertheless, it clearly emerges how innovation policies promoting OI have been largely used by all the countries in the sample, representing an empirical validation of recent conceptual studies supporting OI’s strategic role during emergency (e.g., Chesbrough, 2020).

### 6.2 Implications for policymakers

Our study provides more evidence that innovation policies that support and promote OI are considered effective by governments to mobilize knowledge and technological resources to develop innovations quickly and in short time periods. Considering the types of innovation policies promoting OI activated by governments worldwide, we find that some instruments are more effective than others in generating and stimulating innovation for faster emergency management response. We provide further evidence on government behaviors during the emergency versus the normal period, focusing the attention on what policy instruments had greater usage during the Covid‐19 period compared with normal times (i.e., formal consultation with stakeholders and experts; fellowships and postgraduate loans and scholarships; networking and collaborative platforms; dedicated support to research infrastructures).

As a final word, there is no doubt this period has seen some remarkable innovations from governments worldwide, and we hope our comprehensive analysis of secondary data offers new insights on how quickly behaviors and structures can change to ensure that the importance of collaboration and innovation are optimized to create a lasting impact on society.

### 6.3 Limitations and future research opportunities

The authors recognize the study has some limitations, which opens up opportunities for future developments.

First, this research is exploratory in nature, and the data analysis techniques used, i.e., cluster analyses and ANOVA can only provide differences between groups and moments of time, but they cannot be used to establish causal relationships. Further research can be focused on exploring in a more robust way the path‐dependency between innovation policy strategies during normal times and during emergency situations.

Second, by using the OECD data, we needed to accept the OECD classification of policy instruments that, for some groups, could be considered quite broad (e.g., in the case of financial support instruments). Further research could decide to adopt a different classification of innovation policies and/or break down more the unit of analysis. Connected to this, using the OECD data limits the sample size to the number of OECD countries. Including non‐OECD countries could enrich the findings and also improve the robustness of the analyses.

Finally, this paper offers fresh insights by examining an important snap shot in time and the reaction that governments took at the beginning of this unique global emergency in the ‘modern’ world. On the one hand, this exposes the relevance of the highly regulated but modern environment but, on the other hand, the research exposes the extraordinary measures taken during a specific and unique event. Since the emergency is ongoing, it is not yet possible to assess the long‐term effectiveness of these policy interventions' which should be assessed through future studies. So, it will be interesting to explore if and how the cluster compositions – representing the attitude of different countries regarding collaborative innovation – might change even more after governments have experienced the benefits of using OI practices on a longer‐term. Further research assessing the full impact of these policies after the Covid‐19 crisis period could also provide an interesting point of reflection for mitigating the impact of future emergencies.

## Author contributions

All the authors contributed to the research and paper development.

## Conflict of interest

There is no conflict of interest.

## Notes

^1^

https://wirvsvirushackathon.org/?lang=en

^2^

https://www.dfg.de/foerderung/info_wissenschaft/2020/info_wissenschaft_20_20/index.html

^3^

https://www.csis.org/analysis/russias‐response‐covid‐19

^4^

https://oecd‐opsi.org/covid‐response/epidemiological‐reporting‐system‐ems‐a‐register‐according‐to‐the‐epidemic‐law‐%c2%a74/

^5^

https://oecd‐opsi.org/covid‐response/homecare‐app/

^6^

https://sdg.iisd.org/commentary/policy‐briefs/event‐discusses‐costa‐ricas‐efforts‐in‐achieving‐sustainable‐economic‐recovery‐from‐covid‐19/

^7^

https://www.china‐briefing.com/news/chinas‐latest‐regional‐measures‐to‐support‐smes‐during‐coronavirus‐outbreak/

^8^

https://oecd‐opsi.org/covid‐response/national‐institutes‐of‐health‐launch‐public‐private‐partnership‐to‐speed‐covid‐19‐vaccine/

^9^

https://www.zdnet.com/article/covid‐19‐australias‐new‐app‐whatsapp‐chat‐and‐telehealth‐launch/

^10^

https://play.google.com/store/apps/details?id=br.gov.datasus.guardioes&hl=en_US

^11^

https://www.gov.uk/government/news/billion‐pound‐support‐package‐for‐innovative‐firms‐hit‐by‐coronavirus

^12^

https://oecd‐opsi.org/covid‐response/greecevsvirus‐digital‐innovation‐against‐covid‐19‐2/

^13^

https://oecd‐opsi.org/covid‐response/inov‐4‐covid‐19/

^14^

https://oecd‐opsi.org/covid‐response/doctorates‐4‐covid‐19/

^15^

https://oecd‐opsi.org/covid‐response/portugals‐collaborative‐office‐for‐digital‐response‐to‐covid‐19/

## Appendix A OECD survey on the Science, Technology and Innovation policy responses to Covid‐19 (https://stip.oecd.org/Covid.html)

The OECD STIP survey seeks to collect information on STI policy measures to respond to Covid‐19 and intend to provide a cross‐country information service that S&I policymakers can use when designing their own policies.

Survey questions are organized in the following four areas:

1. Scientific advice and communication
  - What arrangements do you have in place to ensure scientific advice informs national policy and decision making in relation to Covid‐19?
  - In what ways are you coordinating on Covid‐19 STI responses at the international level?
  - Do you have dedicated arrangements in place for communicating science advice and for refuting misleading information to the public on Covid‐19?
2. Collaboration mechanisms
  - At the national level, what mechanisms are you developing or relying upon to bring together different STI actors (researchers, industry, government, health sector, foundations, etc.) to effectively collaborate on responses to Covid‐19?
  - At the international level, what mechanisms are you developing or relying upon to bring together different STI actors (researchers, industry, government, health sector, foundations, etc.) to effectively collaborate on responses to Covid‐19?
3. Specific or novel measures
  - What new STI policy measures is your country taking to respond specifically to the Covid‐19 crisis?
  - What novel approaches is your country using to address the coronavirus crisis (e.g., use of machine learning, open science initiatives boosting access and sharing of data and research results, development and use of prediction models, etc.)?
4. Impacts on the system
  - What impact on the STI system do you anticipate in the short‐, medium‐ and long‐term, and what measures are you implementing to address those?
  - Is support of the STI system part of planned stimulus packages aimed at supporting the economy?
  - Is there anything else regarding the STI policy response to Covid‐19 in your country you would like to mention?

## Appendix B Innovative Covid‐19 response by governments and public interest organizations (https://oecd‐opsi.org/covid‐response/)

The OECD Open and Innovative Government Division and the Centre for Public Impact are issuing this call to all levels of government, civil society, international organizations, and the private sector to gather innovative solutions and inspiration on how individuals and organizations across the globe are responding to the crisis.

Please answer the following questions:

- What is the innovative response?
  ◦ Describe the key aspects. What is important to know about it?
- What general issue(s) is this addressing?^*^
  ◦ Patient Care
  ◦ Health and Safety of Responders
  ◦ Information and practice sharing (with public and/or internal)
  ◦ Resource management and mobilization
  ◦ Governance responses
  ◦ Real‐time data collection, sharing, and analysis
  ◦ Public service delivery under new circumstances
  ◦ Crowdsourcing solutions
  ◦ Social effects of the crisis
  ◦ Add new choice: _________________________
- What specific issue is this solution intended to address? What is the anticipated or expected impact?
- Give the innovative response a short title
- Organizations/institutions involved (list all that play a role).
- Please select the level(s) of government most relevant to the innovation
  ◦ National/Federal Government
  ◦ Regional/State Government
  ◦ Local Government
  ◦ International Organization
  ◦ Private Sector
  ◦ Non‐profit/Civil Society
  ◦ Other: _________________
- Primary URL (Link to innovation or related documentation – if available): _________________________
- Country: _______________________
- Your email for questions/clarification: _________________________

## References

[radm12495-bib-0002] Ahn, J.M. , Roijakkers, N. , Fini, R. , and Mortara, L. (2019) Leveraging Open Innovation to improve society: past achievements and future trajectories. R&D Management, 49, 3, 267–278.

[radm12495-bib-0004] Azoulay, P. and Jones, B. (2020) Beat COVID‐19 through innovation. Science, 368, 6491, 553. 32381693 10.1126/science.abc5792

[radm12495-bib-0005] Beck, S. , Bergenholtz, C. , Bogers, M. , Brasseur, T.‐M. , Conradsen, M.L. , Di Marco, D. , Distel, A.P. , Dobusch, L. , Dörler, D. , Effert, A. , Fecher, B. , Filiou, D. , Frederiksen, L. , Gillier, T. , Grimpe, C. , Gruber, M. , Haeussler, C. , Heigl, F. , Hoisl, K. , Hyslop, K. , Kokshagina, O. , LaFlamme, M. , Lawson, C. , Lifshitz‐Assaf, H. , Lukas, W. , Nordberg, M. , Norn, M.T. , Poetz, M. , Ponti, M. , Pruschak, G. , Pujol Priego, L. , Radziwon, A. , Rafner, J. , Romanova, G. , Ruser, A. , Sauermann, H. , Shah, S.K. , Sherson, J.F. , Suess‐Reyes, J. , Tucci, C.L. , Tuertscher, P. , Vedel, J.B. , Velden, T. , Verganti, R. , Wareham, J. , Wiggins, A. , and Xu, S.M. . (2020) The Open Innovation in Science research field: a collaborative conceptualisation approach. Industry and Innovation, , 1–50.

[radm12495-bib-0006] Bogers, M. , Chesbrough, H. , and Moedas, C. (2018) Open Innovation: research, practices, and policies. California Management Review, 60, 2, 5–16.

[radm12495-bib-0007] Bommert, B. (2010) Collaborative innovation in the public sector. International Public Management Review, 11, 1, 15–33.

[radm12495-bib-0008] Chesbrough, H.W. (2003) Open Innovation: The New Imperative for Creating and Profiting from Technology. Boston, MA: Harvard Business Press.

[radm12495-bib-0009] Chesbrough, H. (2020) To recover faster from Covid‐19, open up: managerial implications from an Open Innovation perspective. Industrial Marketing Management, 88, 410–413.

[radm12495-bib-0010] Chesbrough, H. and Bogers, M. (2014) Explicating open innovation: clarifying an emerging paradigm for understanding innovation. In: Chesbrough, H. , Vanhaverbeke, W. , and West, J. (eds), New Frontiers in Open Innovation, Oxford: Oxford University Press. pp. 3–28.

[radm12495-bib-0011] Chesbrough, H. and Brunswicker, S. (2013) Managing Open Innovation in Large Firms. Garwood Center for Corporate Innovation at California University, Berkeley in US & Fraunhofer Society in Germany.

[radm12495-bib-0012] Chesbrough, H. and Crowther, A.K. (2006) Beyond high tech: early adopters of Open Innovation in other industries. R&D Management, 36, 3, 229–236.

[radm12495-bib-0051] Chesbrough, H. , and Di Minin, A. (2014) Open social innovation. New Frontiers in Open Innovation, 16, 301–315.

[radm12495-bib-0013] Chesbrough, H. and Schwartz, K. (2007) Innovating business models with co‐development partnerships. Research‐Technology Management, 50, 1, 55–59.

[radm12495-bib-0014] Chesbrough, H.W. and Vanhaverbeke, W. (2018) Open innovation and public policy in the EU with implications for SMEs. In: Vahaverbeke, W. (Ed.), Researching open innovation in SMEs. Singapore: World Scientific. pp. 455–492.

[radm12495-bib-0015] Cusumano, M.A. , Gawer, A. , and Yoffie, D.B. (2019) The Business of Platforms: Strategy in the Age of Digital Competition, Innovation, and Power. New York: Harper Business.

[radm12495-bib-0016] De Jong, J.P. , Kalvet, T. , and Vanhaverbeke, W. (2010) Exploring a theoretical framework to structure the public policy implications of Open Innovation. Technology Analysis & Strategic Management, 22, 8, 877–896.

[radm12495-bib-0017] De Silva, M. and Wright, M. (2019) Entrepreneurial co‐creation: societal impact through Open Innovation. R&D Management, 49, 3, 318–342.

[radm12495-bib-0018] EC/OECD . (2020a) STIP Compass Taxonomies Describing STI Policy Data, edition 2019. https://stip.oecd.org.

[radm12495-bib-0019] EC/OECD . (2020b) STIP Compass: International Database on Science, Technology and Innovation Policy (STIP), edition 1/1/2020. https://stip.oecd.org.

[radm12495-bib-0020] Edquist, C. (2001) The systems of innovation approach and innovation policy: an account of the state of the art. Paper presented at the DRUID Conference, Aalborg.

[radm12495-bib-0021] Enkel, E. , Bogers, M. , and Chesbrough, H. (2020) Exploring Open Innovation in the digital age: a maturity model and future research directions. R&D Management, 50, 1, 161–168.

[radm12495-bib-0022] Felin, T. and Zenger, T.R. (2014) Closed or Open Innovation? Problem solving and the governance choice. Research Policy, 43, 5, 914–925.

[radm12495-bib-0023] Gascó, M. (2017) Living labs: implementing Open Innovation in the public sector. Government Information Quarterly, 34, 1, 90–98.

[radm12495-bib-0024] George, G. , Rao‐Nicholson, R. , Corbishley, C. , and Bansal, R. (2015) Institutional entrepreneurship, governance, and poverty: insights from emergency medical response services in India. Asia Pacific Journal of Management, 32, 1, 39–65.

[radm12495-bib-0026] Jugend, D. , Fiorini, P.D.C. , Armellini, F. , and Ferrari, A.G. (2020) Public support for innovation: a systematic review of the literature and implications for Open Innovation. Technological Forecasting and Social Change, 156, 119985.

[radm12495-bib-0027] Kankanhalli, A. , Zuiderwijk, A. , and Tayi, G.K. (2017) Open Innovation in the public sector: a research agenda. Government Information Quarterly, 34, 1, 84–89.

[radm12495-bib-0049] Laperche, B. , Lefebvre, G. , and Langlet, D. (2011) Innovation strategies of industrial groups in the global crisis: Rationalization and new paths. Technological Forecasting and Social Change, 78, 8, 1319–1331.

[radm12495-bib-0028] Leckel, A. , Veilleux, S. , and Dana, L.P. (2020) Local open innovation: a means for public policy to increase collaboration for innovation in SMEs. Technological Forecasting and Social Change, 153, 119891.

[radm12495-bib-0029] Lee, S.M. , Hwang, T. , and Choi, D. (2012) Open Innovation in the public sector of leading countries. Management Decision, 50, 1, 147–162.

[radm12495-bib-0030] Mejia, J. , Urrea, G. , and Pedraza‐Martinez, A.J. (2019) Operational transparency on crowdfunding platforms: effect on donations for emergency response. Production and Operations Management, 28, 7, 1773–1791.

[radm12495-bib-0031] Mergel, I. and Desouza, K.C. (2013) Implementing open innovation in the public sector: the case of Challenge.gov. Public Administration Review, 73, 6, 882–890.

[radm12495-bib-0032] OECD . (2021) STIP COVID‐19 Watch. https://stip.oecd.org/covid/

[radm12495-bib-0033] Okazaki, S. (2006) What do we know about mobile Internet adopters? A cluster analysis. Information & Management, 43, 2, 127–141.

[radm12495-bib-0034] OPSI . (2020) OPSI . COVID‐19 Innovative Response Tracker. https://oecd‐opsi.org/covid‐response/

[radm12495-bib-0035] Park, C.H. and Johnston, E.W. (2018) An event‐driven lens for bridging formal organizations and informal online participation: how policy informatics enables just‐in‐time responses to crises. In: Gil‐Garcia, J.R. , Pardo, T.A. , and Luna‐Reyes, L.F. (Eds.), Policy Analytics, Modelling, and Informatics. Cham: Springer, pp. 343–361.

[radm12495-bib-0036] Pedersen, K. (2020) What can Open Innovation be used for and how does it create value? Government Information Quarterly, 37, 2, 101459.

[radm12495-bib-0037] Pisano, G.P. and Verganti, R. (2008) What kind of collaboration is right for you. Harvard Business Review, 86, 12, 78–86.

[radm12495-bib-0038] Randhawa, K. , Wilden, R. , and West, J. (2019) Crowdsourcing without profit: the role of the seeker in open social innovation. R&D Management, 49, 3, 298–317.

[radm12495-bib-0039] Rayna, T. and Striukova, L. (2019) Open social innovation dynamics and impact: exploratory study of a fab lab network. R&D Management, 49, 3, 383–395.

[radm12495-bib-0040] Rousseeuw, P.J. (1987) Silhouettes: a graphical aid to the interpretation and validation of cluster analysis. Journal of Computational and Applied Mathematics, 20, 53–65.

[radm12495-bib-0041] Shepherd, D.A. and Williams, T.A. (2014) Local venturing as compassion organizing in the aftermath of a natural disaster: the role of localness and community in reducing suffering. Journal of Management Studies, 51, 6, 952–994.

[radm12495-bib-0042] Smart, P. , Holmes, S. , Lettice, F. , Pitts, F.H. , Zwiegelaar, J.B. , Schwartz, G. , and Evans, S. (2019) Open Science and Open Innovation in a socio‐political context: knowledge production for societal impact in an age of post‐truth populism. R&D Management, 49, 3, 279–297.

[radm12495-bib-0043] Sørensen, E. and Torfing, J. (2011) Enhancing collaborative innovation in the public sector. Administration & Society, 43, 8, 842–868.

[radm12495-bib-0044] UNECE . (2017) Innovation in the Public Sector: Country Experiences and Policy Recommendations. New York and Geneva: United Nations Publications. ISBN 978‐92‐1‐117141‐9.

[radm12495-bib-0045] Wang, Y. , Vanhaverbeke, W. , and Roijakkers, N. (2012) Exploring the impact of Open Innovation on national systems of innovation – a theoretical analysis. Technological Forecasting and Social Change, 79, 3, 419–428.

[radm12495-bib-0047] Williams, T.A. and Shepherd, D.A. (2016) Building resilience or providing sustenance: different paths of emergent ventures in the aftermath of the Haiti earthquake. Academy of Management Journal, 59, 6, 2069–2102.

[radm12495-bib-0048] Yun, J.J. , Zhao, X. , and Hahm, S.D. (2018) Harnessing the value of open innovation: Change in the moderating role of absorptive capability. Knowledge Management Research & Practice, 16, 3, 305–314.

[radm12495-bib-0050] Zouaghi, F. , Sánchez, M. , and Martínez, M.G. (2018) Did the global financial crisis impact firms' innovation performance? The role of internal and external knowledge capabilities in high and low tech industries. Technological Forecasting and Social Change, 132, 92–104.

